# Outcomes after anterior cervical discectomy and fusion for presumed segmental dysfunction-related neck pain: a Swedish nationwide registry study

**DOI:** 10.1007/s00701-026-07076-y

**Published:** 2026-10-22

**Authors:** Ahmed Haydar Sameer, Zehra Nazar, Victor Gabriel El-Hajj, Erik Edström, Paul Gerdhem, Adrian Elmi-Terander, Victor E. Staartjes

**Affiliations:** 1 Department of Clinical Neuroscience, Karolinska Institutet, Stockholm, Sweden; 2 Machine Intelligence in Clinical Neuroscience & Microsurgical Neuroanatomy (MICN) Laboratory, Department of Neurosurgery, Clinical Neuroscience Center, Zurich, Switzerland; 3 Capio Spine Center Stockholm, Löwenströmska Hospital, Upplands Väsby, Sweden; 4 Department of Surgical Sciences, Uppsala University, Uppsala, Sweden; 5 Department of Orthopaedics and Hand Surgery, Uppsala University Hospital, Uppsala, Sweden; 6 Department of Clinical Science, Intervention and Technology, Karolinska Institutet, Stockholm, Sweden; 7 Department of Medical Sciences, Örebro University, Örebro, Sweden

**Keywords:** Anterior discectomy and fusion, Dysfunctional cervical segment, Neck pain, Patient reported outcome measures, Swespine, MCID

## Abstract

**Purpose:**

Surgical management of mechanically driven neck pain without radiculopathy or myelopathy is a controversial topic, where comparative evidence to non-operative care is limited. This registry-based descriptive cohort study, without a nonoperative comparator, aimed to evaluate longitudinal outcomes after anterior cervical discectomy and fusion (ACDF) for mechanically driven pain presumed to derive from a dysfunctional cervical segment.

**Methods:**

This observational cohort included all patients identified in Swespine (2006–2024) who underwent ACDF for presumed segmental dysfunctional-related neck pain and had an available baseline NRS neck pain value, and at least one postoperative NRS neck pain follow-up value. Outcome measures included NRS neck pain, Neck Disability index (NDI), health-related quality-of-life index (EQ5D), and postoperative satisfaction. PROMs were observed at 1, 2, 5, and 10 years postoperatively. Clinically meaningful improvements were defined through established MCID thresholds: ≥ 40% relative reduction in NRS, ≥ 33% relative reduction in NDI, and ≥ 0.08 increase in EQ5D.

**Results:**

A total of 36 patients met the inclusion criteria. Mean age was 51 ± 10 years and 50% were males. PROMs at 1-year follow-up revealed MCID achievement in 55% (18/33) for NRS neck pain, 48% (15/31) for NDI, 53% (16/30) for EQ-5D and 64% (21/33) for patient satisfaction following surgery. The improvement of PROMs was predominantly observed at 1 year, with no clear additional improvement thereafter. Patient satisfaction remained consistently high at all time points, ranging from 64–71%.

**Conclusions:**

Despite likely substantial selection bias in this group, ACDF was followed by MCID attainment in roughly half of the patients, at one year postoperatively. Patient satisfaction remained relatively high despite heterogeneous PROM trajectories. The results do not support recommendations for surgical treatment in this patient group. Because of the absence of controls, small sample size, and registry-defined diagnosis, causality cannot be inferred. Prospective studies with well-defined, objective, inclusion criteria are warranted to investigate the possible effects of surgical treatment for neck pain alone.

**Supplementary Information:**

The online version contains supplementary material available at https://doi.org/10.1007/s00701-026-07076-y.

## Introduction

Anterior cervical discectomy and fusion (ACDF) is a commonly performed procedure in spinal surgery worldwide and is well established for the treatment of cervical radiculopathy or myelopathy [22, 31]. For these indications, previous studies have shown effective and rapid improvements in pain, neurological symptoms, and functionality [22]. However, to our knowledge, no studies have evaluated the impact of ACDF in the subgroup of patients with presumed segmental dysfunction-related neck pain (hereafter referred to as segmental neck pain).

Neck pain is a highly prevalent condition, contributing largely to disability and population burden [14]. The etiology is multifactorial, which highlights that the term “neck pain” is not a single entity, but rather a heterogeneous umbrella term [18]. Within this spectrum, mechanical or movement-provoked neck pain typically refers to pain triggered by spinal motion or loading, with the absence of myelopathic or radiculopathic symptoms [1, 28]. In Swedish clinical practice, the label segmental pain (Segmentell rörelsesmärta) is used to describe a narrow subgroup of mechanically induced pain that is presumed to derive from a dysfunctional segment [34, 35, 37]. The diagnosis includes patients with a history of mechanically provoked pain, imaging evidence of degenerative changes, and clinical findings of level-specific pain that is provoked by deep palpation that corresponds to the radiologic level of degeneration. The literature proposes several potential pain generators that may contribute to the mechanically provoked pain, including degenerative changes, discogenic pain, ligament, and facet joint wear [15, 26, 34, 35, 37]. However, imaging findings are common and do not automatically identify the pain generator. Thus, even when the pain is motion-provoked and level-specific, pain generators cannot be reliably distinguished, which is why surgery for isolated neck pain remains controversial. Consequently, the identification of patients with segmental neck pain heavily relies on the surgeon’s clinical assessment of the primary pain generator [35]. Moreover, there are currently no standardised objective diagnostic criteria that reliably distinguish segmental neck pain from broader categories of neck pain, or that allow safe distinction of anatomical pain generators (disc, facet joints, or myalgia). Segmental neck pain is not commonly used as a diagnostic term in international neck pain literature, possibly due to the lack of description of distinct symptoms that might be associated with the dysfunctional segment [26]. Instead, similar presentations in the literature are described using broader terms such as mechanical neck pain, non-specific neck pain or axial neck pain [3, 16].

Segmental pain has been more extensively applied in the lumbar spine, where fusion may be considered in carefully selected patients after conservative attempts are inadequate, although evidence is ambiguous. The supporting evidence for surgery remains thin, and the indication is debated [25]. While the impact of surgery is reported to improve outcomes in selected patients with severe chronic or axial lower back pain [12, 20], reviews of randomised long-term data have been interpreted to show no superiority over conservative care in terms of pain and disability [21]. Although segmental pain has predominantly been applied in the lumbar spine, it has also been used to describe a similar but smaller subgroup of patients in the cervical spine and attempts to translate similar surgical principles have been made.

Cervical surgery for neck pain presumed to be driven by the dysfunctional segment has been less studied, largely because interventions have been avoided in the absence of spinal instability, neurological deficits, or severe deformities [5, 33]. Non-operative management is suggested to benefit most patients, while comparative evidence demonstrating superior outcomes with surgery remains limited [39]. Several case series report significant improvements following surgery for axial neck pain, however, these findings are often derived from uncontrolled studies [13, 27, 30, 32]. Additionally, a systematic review identified no available comparative evidence to non-operative care [32]. In patients undergoing ACDF for other surgical indications, such as cervical radiculopathy or myelopathy, postoperative reductions in neck pain have been reported, where 7% to 20% experience complete resolution, and 54% to 64% experience partial resolution of neck pain at 1 year postoperatively, suggesting that axial neck pain may improve after ACDF even when it is not the primary surgical indication [6]. However, the findings do not establish any effect of ACDF in patients treated for motion-provoked pain.

To date, there is no published evidence that explicitly addresses segmental neck pain, and the absence of standardised classifications further complicates case identification. Consequently, outcomes after surgery for neck pain presentations similar to segmental neck pain remain inconsistent, the evidence base limited, and the role of ACDF for this population remains unexplored. In the light of this gap, the following study reviews the national Swedish experience of ACDF in the subgroup of patients with segmental neck pain using data derived from Swespine Registry [23]. The aim of the study was to describe longitudinal PROM changes and satisfaction rates following ACDF in this unexplored patient group.

## Methods

### Patient selection

The study's intention was to identify and analyse all patients with segmental neck pain, which describes a condition where the presumed source of the pain derives from a dysfunctional segment of the cervical spine. Therefore, patients were selected based on the surgeon-reported diagnosis “Segmentell rörelsesmärta” found in Swespine registry that describes a mechanically induced pain, presumed to derive from a dysfunctional segment.

The operational case definition of segmental neck pain was ACDF performed in patients registered in Swespine with “Segmentell rörelsesmärta”, meaning “Segmental pain upon movement”. The registry diagnosis was available as a diagnostic option throughout the study period. The diagnosis was recorded in the Swespine variable OpHRDiagnos1 (coding value 4) and was recorded as the primary surgical indication in all included cases. Detailed diagnostic elements, including preoperative MRI findings, facet arthropathy, Modic changes, dynamic radiographs, provocative discography, diagnostic injections, and instability parameters were not available as structured, extractable variables in the Swespine dataset. The cohort was therefore defined by the surgeon-reported registry diagnosis rather than by independently verifiable diagnostic criteria.

The study reviewed prospectively recorded data of all patients who underwent ACDF for segmental neck pain identified in the nationwide Swespine registry. All adult patients surgically treated between January 2006 and December 2024 who had an available baseline NRS neck pain value and at least one postoperative NRS neck pain follow-up value at any given timepoint were eligible for inclusion. Cases with no NRS neck pain baseline or no postoperative follow-up value of NRS neck pain were excluded. Missing baseline or follow-up values of outcome variables other than NRS neck pain did not cause exclusion. Each outcome was analysed on a complete-case basis, meaning that patients were included in each PROM analysis only if they had both baseline and corresponding follow-up data for that specific outcome. This study was conducted in line with the STROBE guidelines [4].

### Data source

Swespine is a national quality registry in Sweden that prospectively collects standardised preoperative and postoperative data for patients undergoing spinal surgery for degenerative spinal conditions. The registry covers approximately 95% of spine surgery units across the country and includes both demographic data as well as patient reported outcomes after spinal surgery [2, 9, 10, 36, 38]. As of 2023, Swespine included data from about 50 spine centers and had a coverage of 95%, which contained a total of approximately 192,800 index surgeries registered. For cervical spine surgery specifically, 2024 Swespine annual report show that approximately 26–27 centres reported 1,641 cervical procedures nationally for 2023, and that the national 1-year follow-up rate for cervical procedures performed in 2022 ranged from 59 to 66%, depending on diagnosis [23].

### Variables

Demographic variables included age, sex, body mass index (BMI), smoking status, prior cervical surgery, and preoperative analgesic medication use. Analgesic use was defined as patient-reported regular use of analgesic medication before surgery, as recorded in Swespine, and was analysed as a binary yes/no variable. Prior cervical surgery was available as a binary yes/no variable, in which the type of prior cervical surgery was not recorded as structured Swespine variables. Surgical and perioperative variables included the number of operated levels and length of hospital stay. Procedural complications were defined as operation-related complications reported on the day of surgery. Reoperations after the index ACDF procedure were also recorded. These variables were reported descriptively to provide clinical context for the study cohort. Missing values are reported in the corresponding tables.

Several variables used to characterise ACDF candidates were unavailable in Swespine as structured data. These included detailed imaging findings, sagittal alignment, facet degeneration, number of degenerated levels, flexion–extension instability, prior nonoperative care, mental health-related measures, work status, symptom duration, and dominant pain pattern.

The PROMs used in the study included: EQ5D, Numeric Rating Scale (NRS) for neck pain, Neck Disability Index (NDI), and postoperative satisfaction. The EQ-5D instrument evaluates health status across five aspects: mobility, self-care, usual activities, pain/discomfort and anxiety/depression, each of them graded on three severity levels [29]. The three levels are: no problems, moderate problems, or severe problems. The answers are then merged to provide a single index value, EQ-5D-3L_Index_, that can be used to evaluate overall health. The index value runs from – 0.59 (worst) to 1.0 (best) and the Swedish EQ-5D-3L value set was used in the study [17]. NRS for neck pain is a numerical pain rating measure that assesses pain intensity by asking patients to rate their neck or arm pain on a scale from 0 (no pain) to 10 (worst possible pain) [11]. NDI is a questionnaire that quantifies information on how a patient’s neck pain affects their different aspects of daily function [40]. The questionnaire is divided into 10 different questions of everyday activities. Postoperative satisfaction was measured by a single question in which patients expressed their postoperative satisfaction as either being satisfied, undecided, or unsatisfied. For analysis, patient satisfaction was dichotomised as satisfied versus not satisfied; the “undecided” and “dissatisfied” responses were grouped as not satisfied. The raw three-category satisfaction responses were also reported separately at each follow-up time point.

### Evaluation of clinical success

To evaluate clinical success of the surgical approach, the percentage of patients who reached minimally clinically important difference (MCID) after 1-, 2-, 5- and 10-years compared to baseline was calculated for the analysed PROMs. The cutoff points for the smallest difference reflecting clinically important outcomes were ≥ 40% relative reduction in NRS neck pain intensity, ≥ 33% relative reduction in NDI scores, and a ** ≥ **0.08 absolute increase in EQ5D index [8]. The selected responder definitions are in line with recent cervical PROM work, which supports relative change for NRS and NDI and absolute change for EQ-5D [7, 8]. The defined thresholds are based on previously published literature on cervical spine surgery on broader degenerative cervical spine surgery populations. In the absence of MCID thresholds validated specifically for isolated non-radicular neck pain, these thresholds were applied as the most relevant available benchmarks. As a sensitivity analysis, absolute thresholds were applied where possible: ≥ 3 point reduction for NRS neck pain and a ≥ 30 point reduction for NDI. EQ-5D is omitted because its primary analysis is already defined by an absolute threshold.

### Statistical analysis

The study used descriptive statistics for all baseline, perioperative, and postoperative variables of the cohort. Continuous variables are presented as means ± standard deviations and categorical variables as counts with percentages. Mean PROM values were presented as time-point specific cross-sectional summaries to illustrate change over time, and medians with interquartile ranges are provided in Supplementary Table S1 for each PROM at each follow-up time point to better understand the distribution of outcomes. A descriptive stratification by prior cervical surgery status was performed for 1-year MCID attainment and satisfaction. MCID attainment proportions, 95% confidence intervals were calculated using the Wilson score method.

Cases of missing data were handled using timepoint-specific complete-case analysis. Follow-up availability was assessed as patients who had reached each postoperative time point by data extraction, regardless of whether they responded, while patients who had not yet reached a given follow-up time point were excluded from the denominator. Supplementary Table S2 reports PROM availability and missingness for each outcome at each follow-up time point.

The missing-data mechanism was unknown. Therefore, best-case and worst-case sensitivity analyses for MCID attainment were performed at 1 and 10 years. No inferential treatment-effect testing was planned or performed; all analyses were descriptive. Mean change from baseline with 95% confidence intervals was calculated among patients with both baseline and corresponding follow-up values at each time point to describe the precision of observed PROM changes. All statistical analyses were conducted with R statistical software (Version 4.5.2, R Foundation for Statistical Computing, Vienna, Austria; RRID:SCR_001905).

### Ethical considerations

Patient inclusion in the Swespine registry follows an opt-out model, therefore, informed consent is not required. Participation in follow-up questionnaires is voluntary.

## Results

**Fig. 1:**
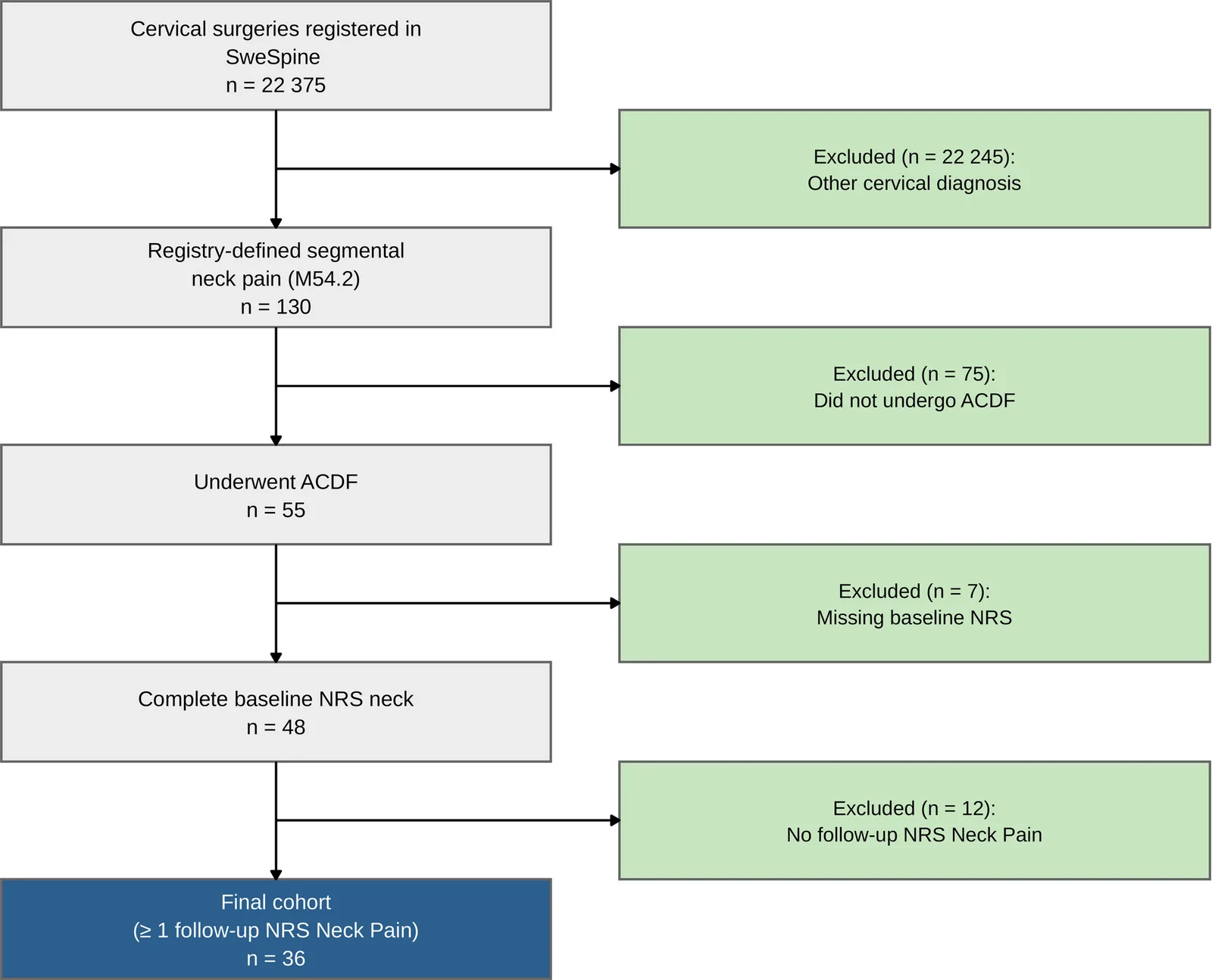
Flowchart illustrating patient inclusion process. Note: ACDF: Anterior Cervical Discectomy and Fusion. NRS, Numeric Rating Scale

A total of 22 375 cervical procedures were identified in Swespine, of which 130 carried the registry-defined diagnosis of segmental neck pain. After excluding 75 non-ACDF procedures, 55 patients remained. The excluded non-ACDF procedures consisted mainly of posterior fixation without decompression (*n* = 48), with the remaining procedures comprising a diverse group of other surgical procedures. Seven patients were excluded because of missing baseline NRS neck pain, and 12 were excluded because of absent postoperative NRS neck pain follow-up, resulting in a final cohort of 36 patients (Fig. 1). The 36 included ACDF cases were contributed by 12 centers, with the largest single clinic contributing 7/36 cases (19%). NRS neck pain was available for 33 patients at 1 year, 30 at 2 years, 27 at 5 years, and 18 at 10 years. Following several dropouts, Supplementary Table S3 provides a descriptive comparison of the baseline characteristics of included (*n* = 36) versus excluded (*n* = 19) patients descriptively. Overall, the groups were broadly similar in age, BMI, sex distribution, prior cervical surgery, and proportion of patients undergoing one, and two-level surgery. Excluded patients appeared to have somewhat lower baseline symptom burden, with lower NRS neck pain (6 ± 3 vs 7 ± 2), lower NDI (48 ± 23 vs 56 ± 13), higher EQ-5D (0.34 ± 0.40 vs 0.25 ± 0.28), and less frequent preoperative analgesic medication use (53% vs 94%). However, data completeness among excluded patients was limited.

### Patient characteristics

The mean age was 51 ± 10 years, and BMI was 25 ± 4 kg/m2 (Table 1). The cohort included 18/36 males (50%), and 4/36 (11%) were active smokers. Preoperatively, analgesic medication use was reported by 33/35 (94%), and previous cervical surgery by 8/35 (23%). Data on surgical levels were available for 34 patients: 20/34 (59%) underwent single-level ACDF, 13/34 (38%) two-level ACDF, and 1/34 (3%) three-level ACDF. Mean baseline PROMs were 7 ± 2 for NRS neck pain (*n* = 36), 56 ± 13 for NDI (*n* = 35), and 0.25 ± 0.28 for EQ5D (*n* = 35). Mean length of hospital stay was 3 ± 2 days. Procedural complications were reported in 1/33 (3%), and reoperations after the index ACDF procedure in 3/36 (8%).

**Table 1:** Patient baseline characteristics

Variable	*n* = 36
Age, years (SD)	51 ± 10
BMI (*n* = 35), kg/m2 (SD)	25 ± 4
Sex, male (%)	18 (50%)
Active smokers, *n* (%)	4 (11%)
Preoperative analgesic medication use (*n* = 35), *n* (%)	33 (94%)
Prior cervical surgery (*n* = 35), n (%)	8 (23%)
Levels operated on (*n* = 34)	
Single-level, *n* (%)	20 (59%)
Two-level, *n* (%)	13 (38%)
Three-level, *n* (%)	1 (3%)
Baseline PROMs	
NRS neck pain (0–10), (SD)	7 ± 2
NDI (0–100) (*n* = 35), (SD)	56 ± 13
EQ-5D index (−0.594 to 1) (*n* = 35), (SD)	0.25 ± 0.28
Length of stay, days (SD)	3 ± 2
Procedural complications (*n* = 33), *n* (%)	1 (3%)
Reoperation, *n* (%)	3 (8%)

*BMI* body mass index, *NRS* Numeric Rating Scale, *NDI* Neck Disability Index, *EQ-5D* EuroQol-5 Dimensions Index

### MCID attainment and patient satisfaction

The proportion of patients achieving MCID for NRS neck pain, NDI, and EQ-5D at each follow-up interval is summarised in Table 2, together with postoperative satisfaction. Across follow-up time points, approximately half of the cohort achieved MCID for the analysed PROMs, while patient satisfaction remained relatively high. At 1 year, 21 patients were satisfied, 9 undecided, and 3 dissatisfied among 33 respondents. Across all follow-up time points, the dissatisfied group remained small (2–4 patients), while the undecided category accounted for most non-satisfied responses (Supplementary Table S4). Most of the observed improvement was already present at 1 year, with no clear additional improvement at later follow-ups. The number of respondents decreased over time, particularly at 10 years, limiting interpretation of long-term estimates.

**Table 2:** Proportion of patients achieving MCID and reporting satisfaction at each postoperative follow-up

Variable	1 year (*n* = 33)	2 years (*n* = 30)	5 years (*n* = 27)	10 years (*n* = 18)
NRS Neck Pain	18/33 (55%) [38–70%]	15/30 (50%) [33–67%]	14/27 (52%) [34–69%]	8/18 (44%) [25–66%]
NDI	15/31 (48%) [32–65%]	12/30 (40%) [25–58%]	12/26 (46%) [29–65%]	10/17 (59%) [36–78%]
EQ5D	16/30 (53%) [36–70%]	17/29 (59%) [41–74%]	14/23 (61%) [41–78%]	10/16 (62%) [39–82%]
Satisfaction Rate	21/33 (64%) [47–78%]	22/31 (71%) [53–84%]	18/26 (69%) [50–83%]	10/17 (59%) [36–78%]

*NRS* Numeric Rating Scale, *NDI* Neck Disability Index, *EQ5D* EuroQol-5 Dimensions Index score, *MCID* thresholds: ≥ 40% reduction in NRS, ≥ 33% reduction in NDI, and ≥ 0.08 increase for EQ5D. Values are k/n (proportion, 95% Wilson CI). PROM missingness at each follow-up is reported in Supplementary Table S2. The n in each column header is the number of patients with NRS neck pain data at that follow-up

Among patients with prior cervical surgery, 4/8 (50%) achieved MCID for NRS neck pain at 1 year, compared with 13/24 (54%) among patients without prior cervical surgery. Corresponding proportions were 3/8 (38%) versus 11/22 (50%) for NDI, 2/6 (33%) versus 13/23 (57%) for EQ-5D, and 5/8 (63%) versus 15/24 (63%) for satisfaction.

Sensitivity analyses using absolute MCID thresholds are presented in Supplementary Table S5, Panel A. Absolute thresholds yielded similar NRS neck pain responder proportions, whereas NDI responder proportions were lower than with the primary relative-threshold definition. This is partly expected because the absolute NDI threshold of ≥ 30 points correspond to a large relative reduction in this cohort, given the mean baseline NDI of 56. Best-case and worst-case sensitivity analyses for missing MCID outcomes are presented in Supplementary Table S5, Panel B.

**Fig. 2:**
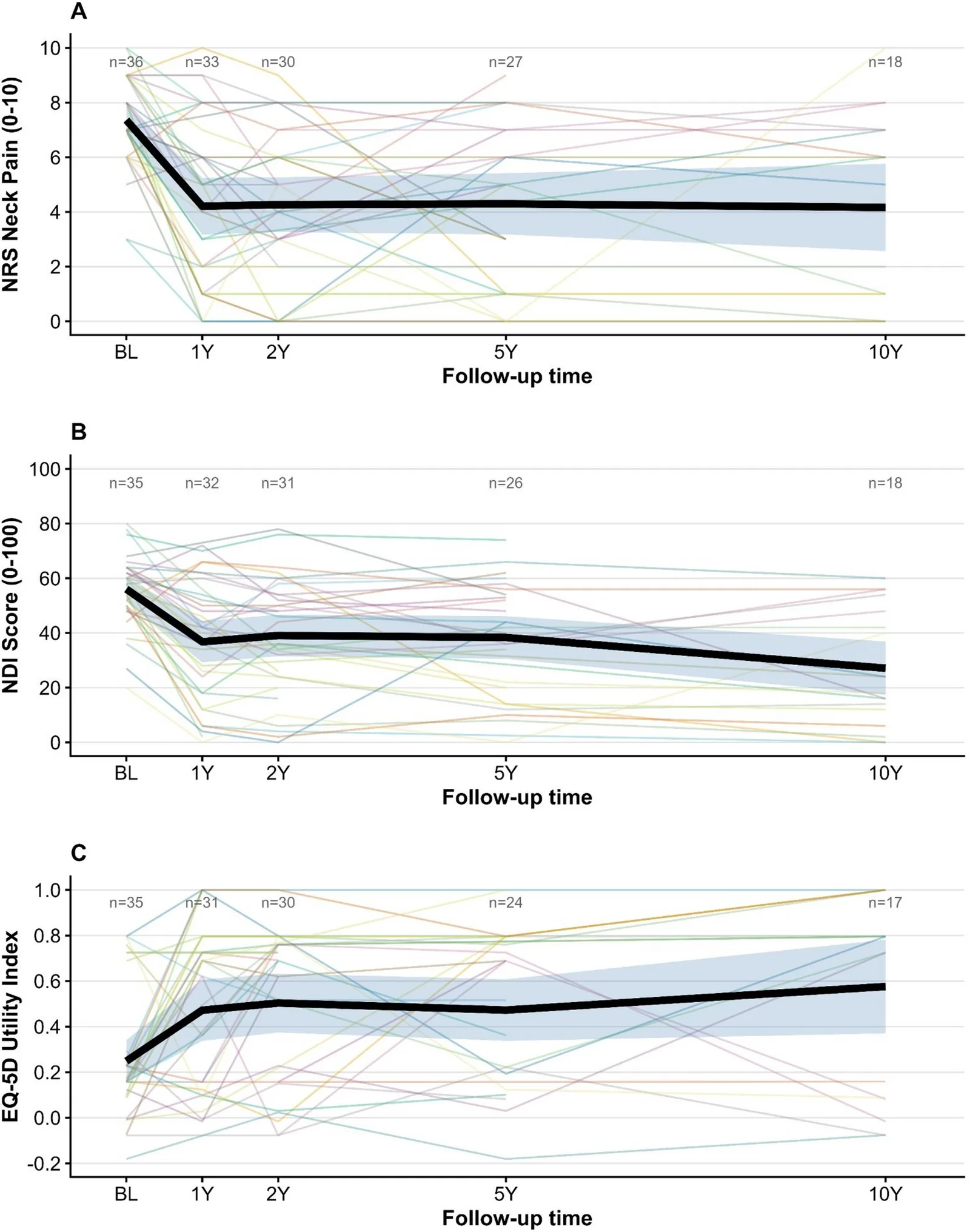
Timepoint-specific cross-sectional summaries. **A** Numeric Rating Scale Neck Pain, (**B**) Neck Disability Index, and (**C**) EuroQol-5 Dimensions Index score at baseline, 1-, 2-, 5-, and 10-year follow-up.The colored lines represent individual patients, and the black line depicts the cohort mean with 95% confidence intervals across all follow-up intervals

Table 3 presents individual PROM trajectories for NRS neck pain, NDI and EQ5D from baseline and through 1-, 2-, 5-, and 10-year follow-up. The table highlights the heterogeneity between patients, where some patients showcase remarkable improvements across all PROMs, while others demonstrate minimal and fluctuating scores over time. Response rates decreased at later follow-up time points, missing responses are displayed as “–”, whereas “NR” indicates that the patient had not yet reached the specified follow-up time point. The same unique patient-level trajectories are visualised in Fig. 2. Mean changes from baseline with 95% confidence intervals are presented in Supplementary Table S6.

**Table 3:** Individual patient-level PROM values at baseline and at 1-, 2-, 5-, and 10 year follow-up

Patient (*n*)	Year of surgery	NRS Neck Pain (0–10)					NDI (0–100)					EQ-5D index (−0.594 to 1)				
		BL	1Y	2Y	5Y	10Y	BL	1Y	2Y	5Y	10Y	BL	1Y	2Y	5Y	10Y
1	2008	9	10	9	1	1	52	66	62	14	0	0.159	0.124	−0.016	0.796	1
2	2007	7	1	0	1	0	58	12	6	8	2	0.228	0.796	0.796	0.76	1
3	2008	9	9	8	8	7	58	60	54	38	54	−0.077	−0.077	−0.077	0.222	−0.077
4	2008	10	5	6	5	1	80	56	34	42	42	0.088	0.691	0.516	0.222	0.725
5	2007	7	1	-	1	-	52	28	-	34	-	0.689	0.796	-	0.796	-
6	2018	9	5	0	5	NR	56	46	26	-	NR	0.725	0.725	0.725	-	NR
7	2013	7	2	2	-	-	54	12	20	-	-	0.157	0.796	0.796	-	-
8	2014	6	4	-	0	0	60	40	-	22	18	0.088	0.689	-	1	-
9	2007	6	0	5	0	10	20	0	10	0	40	0.796	0.088	0.796	0.124	0.088
10	2008	6	5	6	-	6	38	34	36	-	24	0.76	0.362	0.76	-	0.796
11	2011	9	-	2	2	2	48	-	24	12	14	0.228	-	0.76	0.796	0.796
12	2011	9	5	6	8	-	78	42	58	60	-	0.796	0.62	0.516	0.516	-
13	2012	3	0	0	-	0	-	6	4	-	0	0.258	1	1	-	1
14	2014	7	3	4	1	-	60	54	46	44	-	0.157	0.362	0.689	0.362	-
15	2017	7	8	8	8	NR	76	70	76	74	NR	0.228	0.099	0.03	0.101	NR
16	2007	7	3	-	-	6	49	18	36	-	16	0.17	0.727	0.76	-	0.796
17	2013	8	0	-	-	-	54	2	-	-	-	0.099	1	-	-	-
18	2009	8	1	1	1	1	60	26	24	14	12	0.159	0.796	0.796	0.796	1
19	2021	9	7	-	3	NR	62	38	-	20	NR	−0.008	0.028	-	0.796	NR
20	2010	8	-	4	5	7	64	-	60	66	60	−0.181	-	0.024	−0.181	−0.076
21	2012	6	0	0	6	5	27	4	0	44	24	0.796	1	-	0.193	0.796
22	2012	7	-	8	3	-	68	-	78	54	-	0.258	-	−0.239	0.189	-
23	2013	7	6	6	3	-	64	50	50	62	-	−0.074	0.689	0.62	0.689	-
24	2008	7	1	0	0	0	50	6	2	10	6	0.228	1	1	0.796	1
25	2011	6	8	7	8	6	44	66	64	56	56	0.228	0.157	0.157	0.157	0.159
26	2011	6	2	4	9	-	50	24	44	52	-	0.159	0.725	0.691	-	-
27	2011	9	9	5	6	8	48	42	32	36	56	-	0.157	0.76	0.725	−0.016
28	2013	9	4	7	7	8	48	36	60	36	48	0.157	0.62	−0.077	0.689	0.082
29	2013	7	1	3	6	-	64	44	50	40	-	0.293	−0.008	0.151	0.082	-
30	2011	8	5	5	-	-	64	42	38	-	-	−0.005	-	0.76	-	-
31	2012	9	8	8	7	7	66	62	54	58	16	−0.008	0.099	0.228	0.03	0.725
32	2020	8	4	3	7	NR	62	48	48	53	NR	−0.008	−0.291	0.157	0.689	NR
33	2019	5	6	3	5	NR	60	72	52	48	NR	0.124	−0.016	0.62	-	NR
34	2023	7	6	5	NR	NR	62	52	48	NR	NR	0.157	0.157	0.689	NR	NR
35	2022	3	2	3	NR	NR	36	18	16	NR	NR	0.725	0.725	0.725	NR	NR
36	2023	10	8	-	NR	NR	70	-	-	NR	NR	0.157	-	-	NR	NR

*BL* baseline, *NRS* Numeric Rating Scale, *NDI* Neck Disability Index, *EQ-5D* EuroQol-5 Dimensions Index score. Missing data are reported as "-" (patient did not respond at this follow-up despite sufficient time having elapsed). "NR" (not reached) indicates the patient was operated too recently for the given timepoint to have been reached by data extraction (2026)

## Discussion

This nationwide registry-based study aimed to evaluate all patients who underwent ACDF for a small, registry-defined subgroup with segmental neck pain through comparing pre- and postoperative PROMs and satisfaction rates. Our findings demonstrate that, at the group level, roughly half of the patients achieved clinically meaningful improvement in individual PROMs after surgery, with the largest observable changes in pain, disability, and health-related quality of life predominantly occurring within the first postoperative year. Patient satisfaction remained relatively high across all follow-ups, whereas observed improvements in pain and disability were mainly apparent within the first postoperative year and subsequently appeared to plateau, suggesting temporal stabilization among respondents [7, 9]. Interpretation of longer-term trajectories is limited by decreasing response rates, particularly at 10 years.

Patients undergoing surgery for segmental neck pain make up a small population, and the subgroup remains underrepresented in the literature. By presenting individual trajectories of PROMs, patient satisfaction and MCID achievement after every follow-up, our study provides valuable descriptive outcome data on postoperative outcomes following ACDF for segment-related neck pain and may aid physicians in clinical decision-making when encountering similar patients.

To our knowledge, no study has evaluated surgical outcomes that explicitly apply segmental neck pain to patients with neck pain, and in most reports, the etiology of axial and mechanical neck pain is vaguely specified. Our findings are interpreted in the context of prior cervical literature that evaluates related neck pain presentations. In other words, a pain that is mechanically driven and is presumed with similar pain-generating mechanisms. In the closest comparison identified, Riew et al. performed a systematic review which evaluated ACDF for axial neck pain without neurological deficits [32]. The report evaluated changes in pain, function and patient satisfaction. The review identified three publications which met their inclusion criteria, comprising a total of 166 patients, which again highlights the scarcity of available evidence. Across the included studies, the authors reported a mean improvement of approximately 50% in pain scores, improvement of 39–52% in disability index, and patient satisfaction of 56% and 79% in the two studies where satisfactions were reported. Taken together with our results, this can support the suggestion that a subset of patients may truly benefit and achieve clinically significant improvements following surgery, while also emphasizing the heterogeneity of outcomes and the need for comparative data.

Surgically treating mechanically driven neck pain, without radicular or myelopathic symptoms, is often regarded controversial, mainly due to the multifactorial and often unclear etiology of symptoms, and difficulty of isolating a specific pain generator. Surgery is normally intended for nerve decompression, and neck pain is best treated with medication and targeted physiotherapy [19]. Despite this, our data presents that a proportion of patients achieved significant improvement in reported outcomes, however, there still seems to remain a considerable fraction of the group with no or little improvement, and in some cases even worsening of reported symptoms. This heterogeneity in outcomes raises the question of how we could have such divergent outcomes despite the shared indication. One possible explanation is that different baseline demographics or underlying pain generators could play a role, however the Swespine registry does not provide radiographic evidence or detailed patient information to assist and clarify which individuals are associated with beneficial outcomes after surgical treatment. Therefore, the present study supports further work on standardised objective inclusion criteria for segmental neck pain, predictive factors and the development of standardised protocol for patient selection.

Despite the controversy regarding the indication for surgery, the latest Swespine report (1998–2024) shows that surgery for segmental pain in the lumbar spine accounted for 6% of all spinal surgeries in 2024, underscoring that some patients are still selected for surgery regardless of the inconsistent outcomes reported in the literature [23, 24]. From a clinical standpoint, this pattern supports our rationale presented in the introduction, that even when the literature highlights inconsistent outcomes, some surgeons still operate on patients whose symptoms are interpreted as mechanically driven. The present study should be interpreted within the same context. Our findings do not resolve any ongoing controversy, and the current data does not support recommendation for surgery. Rather, they provide observational evidence that cervical surgery is associated with early, clinically significant improvements of PROMs in a subset of patients and may help inform clinical decision-makings when conservative treatment fails.

A notable trend was the discrepancy between patient-reported satisfaction and the inconsistency of NRS and NDI trajectories, with satisfaction rates remaining high even when NRS and NDI did not indicate proportional improvements. Satisfaction is a subjective measure and may reflect expectations, perceived solution to the long-lasting symptoms, or even acceptance of the residual pain. This discrepancy raises the question of whether the outcomes are based on different underlying factors. Neck pain in general could improve naturally over time, which makes it even more difficult to distinguish surgery-related outcomes in contrast to the natural course of symptoms. Therefore, in this context, a placebo effect is possible, highlighting that uncontrolled observational data should be interpreted with caution and perhaps supports the need for future randomised studies.

## Limitations

This study has several limitations, first and foremost the fundamental limitations that are weaved in throughout the paper; the small cohort and the absence of randomised and comparative evidence, which limits the ability to establish causality when interpreting the findings and directly attribute the improvement to surgery rather than to the natural history of the condition or regression to the mean. No prior power calculation was performed; with 36 patients, the study was underpowered for interferential hypothesis testing. All analyses should therefore be interpreted descriptively. In addition, the MCID thresholds used in this study were derived from broader degenerative cervical spine surgery populations and may not fully capture clinically meaningful improvement in patients undergoing ACDF for non-radicular neck pain. Secondly, we mainly focused on early observations due to decreased responses recorded over time, with only 18 respondents at 10 years, therefore the data on long-term follow-ups are thin and should be interpreted with caution. Thirdly, the most important limitation is the lack of objective, standardised inclusion criteria for this indication. The diagnosis is registry-recorded and relies on the surgeon assessment, which may not fully capture the diagnostic work-up of segmental neck pain. The diagnostic elements supporting the diagnoses were not available as structured registry variables, which introduces the possibility of misclassification. Accordingly, the registry data do not allow differentiation between discogenic, facetogenic, myofascial, instability-related, or nonspecific axial pain. Furthermore, Swespine does not record the type, duration, or failure of preoperative conservative treatment as structured variables, and the physiotherapy-before-surgery variable had low completeness in this cohort (4/36). Prior nonoperative management could therefore not be characterised. In addition, there is likely selection bias as patients undergoing ACDF for segmental neck pain represent a highly selected subgroup. Exclusion of patients with missing baseline NRS neck pain or no postoperative PROM follow-up may have introduced additional selection bias. The final cohort may therefore not fully represent all patients undergoing ACDF for segmental neck pain and the direction of this potential bias effect cannot be determined.

## Conclusion

This nationwide registry-based cohort, on ACDF for segmental neck pain, demonstrated that a subset of patients achieved clinically meaningful PROM improvements, but these registry-derived findings do not support recommending surgery for this indication. The main improvement in PROMs was observed within the first postoperative year and appeared to plateau during the subsequent years. Patient satisfaction remained relatively high despite heterogeneous individual PROM trajectories. Given these findings, together with the limitations of a lack of control studies, the constraint of a small cohort, and the lack of objective standardised inclusion criteria, our data derived findings do not support the recommendation of surgery for the indication of segmental neck pain. Future studies should prioritize exploring objective selection criteria to refine patient selection and clarify treatment effectiveness for selected subgroups that may profit from surgery for intractable chronic neck pain presumed to be derived from segmental dysfunction.

## Supplementary Information

Below is the link to the electronic supplementary material.

Supplementary file1 (DOCX 25 KB)

Supplementary file2 (DOCX 28 KB)

Supplementary file3 (DOCX 25 KB)

Supplementary file4 (DOCX 27 KB)

Supplementary file5 (DOCX 32 KB)

Supplementary file6 (DOCX 27.9 KB)

## Data Availability

The data used in this study were obtained from the Swedish national spine registry Swespine and are not publicly available due to patient confidentiality regulations. Researchers may request access to data via the Swedish Spine Society (SweSpine).
