# Supplementary material for: Outcomes after anterior cervical discectomy and fusion for presumed segmental dysfunction-related neck pain: a Swedish nationwide registry study
Source: Acta Neurochir (Wien). 2026 Oct 22;168(1):245. doi: 10.1007/s00701-026-07076-y (PMC13690893; doi:10.1007/s00701-026-07076-y)
Supplement: Supplementary file 1 [file 701_2026_7076_MOESM1_ESM.docx]

## Supplementary Table S1 Median and interquartile range of PROMs at each timepoint

| **PROM** | **Baseline (n = 36)** | **1 year (n = 33)** | **2 years (n = 30)** | **5 years (n = 27)** | **10 years (n = 18)** |
| --- | --- | --- | --- | --- | --- |
| NRS Neck Pain | 7 [7 to 9] | 4 [1 to 6] | 4 [2 to 6] | 5 [1 to 7] | 6 [1 to 7] |
| NDI | 58 [50 to 64] | 41 [18 to 52] | 44 [24 to 54] | 41 [20 to 54] | 21 [12 to 46] |
| EQ-5D index | 0.159 [0.093 to 0.258] | 0.620 [0.112 to 0.762] | 0.689 [0.157 to 0.760] | 0.603 [0.181 to 0.796] | 0.796 [0.088 to 1.000] |
| *Note: Values are median [Q1 to Q3]. NRS, Numeric Rating Scale. NDI, Neck Disability Index. EQ-5D, EuroQol-5 Dimensions Index score. The column n is the number of patients with NRS neck pain data at each timepoint; NDI and EQ-5D have slightly fewer observations at some timepoints (e.g., at 5 years n = 26 for NDI and 24 for EQ-5D)* | | | | | |
