# Supplementary material for: Outcomes after anterior cervical discectomy and fusion for presumed segmental dysfunction-related neck pain: a Swedish nationwide registry study
Source: Acta Neurochir (Wien). 2026 Oct 22;168(1):245. doi: 10.1007/s00701-026-07076-y (PMC13690893; doi:10.1007/s00701-026-07076-y)
Supplement: Supplementary file 2 [file 701_2026_7076_MOESM2_ESM.docx]

## Supplementary Table S2 Per-PROM missingness at baseline and each postoperative follow-up

| **PROM** | **Baseline** | **1 year** | **2 years** | **5 years** | **10 years** |
| --- | --- | --- | --- | --- | --- |
| NRS Neck Pain | 0/36 (0%) | 3/36 (8%) | 6/36 (17%) | 6/33 (18%) | 10/28 (36%) |
| NDI | 1/36 (3%) | 4/36 (11%) | 5/36 (14%) | 7/33 (21%) | 10/28 (36%) |
| EQ-5D index | 1/36 (3%) | 5/36 (14%) | 6/36 (17%) | 9/33 (27%) | 11/28 (39%) |
| Satisfaction | n/a | 3/36 (8%) | 5/36 (14%) | 7/33 (21%) | 11/28 (39%) |
| *Note: Values are number of patients with missing data / number of patients who had reached the timepoint by data extraction (2026). Patients operated too recently to have reached a given timepoint are excluded from that timepoint's denominator. NRS, Numeric Rating Scale. NDI, Neck Disability Index. EQ-5D, EuroQol-5 Dimensions Index score. The Baseline column reports missing baseline assessments out of 36 patients; satisfaction is not assessed at baseline (n/a). Because complete-case MCID analysis (Table 2) requires both a baseline and a follow-up value, a patient missing the baseline is excluded from that PROM's MCID denominator even when a follow-up value exists (e.g., EQ-5D at 2 years: 30 patients reached the timepoint but 29 had a baseline).* | | | | | |
