# Supplementary material for: Outcomes after anterior cervical discectomy and fusion for presumed segmental dysfunction-related neck pain: a Swedish nationwide registry study
Source: Acta Neurochir (Wien). 2026 Oct 22;168(1):245. doi: 10.1007/s00701-026-07076-y (PMC13690893; doi:10.1007/s00701-026-07076-y)
Supplement: Supplementary file 3 [file 701_2026_7076_MOESM3_ESM.docx]

## Supplementary Table S3 Baseline characteristics of included versus excluded patients

| **Variable** | **Included (n = 36)** | **Excluded (n = 19)** |
| --- | --- | --- |
| Age, years (mean ± SD) | 51 ± 10 (n = 36) | 48 ± 13 (n = 19) |
| BMI, kg/m2 (mean ± SD) | 25 ± 4 (n = 35) | 25 ± 4 (n = 12) |
| Sex, Male | 18 / 36 (50%) | 10 / 19 (53%) |
| Active smokers | 4 / 36 (11%) | 0 / 12 (0%) |
| Preoperative analgesic medication use | 33 / 35 (94%) | 10 / 19 (53%) |
| Prior cervical surgery | 8 / 35 (23%) | 2 / 11 (18%) |
| Single-level surgery | 20 / 34 (59%) | 10 / 16 (62%) |
| Two-level surgery | 13 / 34 (38%) | 4 / 16 (25%) |
| Baseline NRS neck pain (mean ± SD) | 7 ± 2 (n = 36) | 6 ± 3 (n = 12) |
| Baseline NDI (mean ± SD) | 56 ± 13 (n = 35) | 48 ± 23 (n = 10) |
| Baseline EQ-5D (mean ± SD) | 0.25 ± 0.28 (n = 35) | 0.34 ± 0.40 (n = 9) |
| *Note: "Excluded" patients had segmental neck pain (M54.2) and ACDF but were dropped due to missing baseline NRS neck (n = 7) or no follow-up PROM (n = 12). n shown in each cell reflects available data per variable.* | | |
