# Supplementary material for: Outcomes after anterior cervical discectomy and fusion for presumed segmental dysfunction-related neck pain: a Swedish nationwide registry study
Source: Acta Neurochir (Wien). 2026 Oct 22;168(1):245. doi: 10.1007/s00701-026-07076-y (PMC13690893; doi:10.1007/s00701-026-07076-y)
Supplement: Supplementary file 4 [file 701_2026_7076_MOESM4_ESM.docx]

## Supplementary Table S4 Satisfaction at each postoperative follow-up

| **Satisfaction response** | **1 year (n = 33)** | **2 years (n = 31)** | **5 years (n = 26)** | **10 years (n = 17)** |
| --- | --- | --- | --- | --- |
| Satisfied | 21 (64%) | 22 (71%) | 18 (69%) | 10 (59%) |
| Undecided | 9 (27%) | 7 (23%) | 4 (15%) | 5 (29%) |
| Dissatisfied | 3 (9%) | 2 (6%) | 4 (15%) | 2 (12%) |
| *Note: Values are presented as counts with percentages.* | | | | |
