# Supplementary material for: Outcomes after anterior cervical discectomy and fusion for presumed segmental dysfunction-related neck pain: a Swedish nationwide registry study
Source: Acta Neurochir (Wien). 2026 Oct 22;168(1):245. doi: 10.1007/s00701-026-07076-y (PMC13690893; doi:10.1007/s00701-026-07076-y)
Supplement: Supplementary file 5 [file 701_2026_7076_MOESM5_ESM.docx]

## Supplementary Table S5 Sensitivity analyses for MCID attainment

### Panel A. Relative versus absolute MCID thresholds

| **PROM and timepoint** | **Relative threshold** | **Absolute threshold** |
| --- | --- | --- |
| NRS neck, 1 year | 18/33 (55%) | 19/33 (58%) |
| NRS neck, 2 years | 15/30 (50%) | 16/30 (53%) |
| NRS neck, 5 years | 14/27 (52%) | 16/27 (59%) |
| NRS neck, 10 years | 8/18 (44%) | 8/18 (44%) |
| NDI, 1 year | 15/31 (48%) | 7/31 (23%) |
| NDI, 2 years | 12/30 (40%) | 6/30 (20%) |
| NDI, 5 years | 12/26 (46%) | 8/26 (31%) |
| NDI, 10 years | 10/17 (59%) | 9/17 (53%) |
| *Note: Panel A. Values are number of patients achieving MCID / number with complete data (%). Relative thresholds were ≥ 40% reduction in NRS neck pain and ≥33% reduction in NDI. Absolute thresholds were ≥3-point reduction in NRS neck pain and ≥30-point reduction in NDI. NRS, Numeric Rating Scale; NDI, Neck Disability Index.* | | |

### Panel B. Best-case and worst-case MCID scenarios, at 1 and 10 years

| **PROM and timepoint** | **Complete-case** | **Worst-case** | **Best-case** |
| --- | --- | --- | --- |
| NRS neck, 1 year (n = 36) | 18/33 (55%) | 50% | 58% |
| NRS neck, 10 years (n = 28) | 8/18 (44%) | 29% | 64% |
| NDI, 1 year (n = 36) | 15/31 (48%) | 42% | 56% |
| NDI, 10 years (n = 28) | 10/17 (59%) | 36% | 75% |
| EQ-5D, 1 year (n = 36) | 16/30 (53%) | 44% | 61% |
| EQ-5D, 10 years (n = 28) | 10/16 (62%) | 36% | 79% |
| *Note: Panel B. Worst-case assumes every patient with a missing outcome did NOT achieve MCID; best-case assumes every missing outcome DID. The denominator (n) for the worst/best-case percentages is all patients who had reached the timepoint by data extraction (2026).* | | | |
