# Supplementary material for: Outcomes after anterior cervical discectomy and fusion for presumed segmental dysfunction-related neck pain: a Swedish nationwide registry study
Source: Acta Neurochir (Wien). 2026 Oct 22;168(1):245. doi: 10.1007/s00701-026-07076-y (PMC13690893; doi:10.1007/s00701-026-07076-y)
Supplement: Supplementary file 6 [file 701_2026_7076_MOESM6_ESM.docx]

## Supplementary Table S6 Mean change from baseline with 95% CI at each timepoint

| **PROM** | **1 year** | **2 years** | **5 years** | **10 years** |
| --- | --- | --- | --- | --- |
| NRS neck pain | -3.1 [-4.0, -2.1] (n=33) | -3.0 [-4.0, -2.0] (n=30) | -3.3 [-4.5, -2.1] (n=27) | -3.3 [-5.1, -1.5] (n=18) |
| NDI | -17.5 [-23.9, -11.1] (n=31) | -15.2 [-21.7, -8.7] (n=30) | -18.5 [-25.8, -11.2] (n=26) | -22.5 [-35.5, -9.4] (n=17) |
| EQ-5D index | +0.21 [+0.05, +0.36] (n=30) | +0.26 [+0.11, +0.40] (n=29) | +0.23 [+0.04, +0.43] (n=23) | +0.36 [+0.12, +0.61] (n=16) |
| *Note: Values are mean change from baseline [95% CI] (n). Negative values indicate improvement for NRS and NDI; positive values indicate improvement for EQ-5D. NRS, Numeric Rating Scale; NDI, Neck Disability Index.* | | | | |
